# Sum-of-Checks: structured reasoning for surgical safety with large vision-language models

**DOI:** 10.1007/s11548-026-03643-x

**Published:** 2026-05-11

**Authors:** Weiqiu You, Cassandra Goldberg, Amin Madani, Daniel A. Hashimoto, Eric Wong

**Affiliations:** 1 Department of Computer and Information Science, University of Pennsylvania, Philadelphia, PA USA; 2 Department of Surgery, University of Toronto, Toronto, ON Canada; 3 Surgical Artificial Intelligence Research Academy, University Health Network, Toronto, ON Canada; 4 Department of Surgery, Perelman School of Medicine, University of Pennsylvania, Philadelphia, PA USA

**Keywords:** Critical View of Safety, Surgical reasoning, Laparoscopic cholecystectomy, Large vision-language models

## Abstract

****Purpose**:**

Accurate assessment of the Critical View of Safety (CVS) during laparoscopic cholecystectomy is essential to prevent bile duct injury, a complication associated with significant morbidity and mortality. While large vision-language models (LVLMs) offer flexible reasoning, their predictions remain difficult to audit and unreliable on safety-critical surgical tasks.

****Methods**:**

We introduce Sum-of-Checks, a framework that decomposes each CVS criterion into expert-defined reasoning checks reflecting clinically relevant visual evidence. Given a laparoscopic frame, an LVLM evaluates each check, producing a binary judgment and justification. Criterion-level scores are computed via fixed, weighted aggregation of check outcomes. We evaluate on the Endoscapes2023 benchmark using three frontier LVLMs, comparing against direct prompting, chain-of-thought, and sub-question decomposition, each with and without few-shot examples.

****Results**:**

Sum-of-Checks improves average frame-level mean average precision by 12–14% relative to the best baseline across all three models and criteria. Analysis of individual checks reveals that LVLMs are reliable on observational checks (e.g., visibility, tool obstruction) but show substantial variability on decision-critical anatomical evidence.

****Conclusion**:**

Structuring surgical reasoning into expert-aligned verification checks improves both accuracy and transparency of LVLM-based CVS assessment, demonstrating that explicitly separating evidence elicitation from decision-making is critical for reliable and auditable surgical AI systems.

## Introduction

**Fig. 1:**
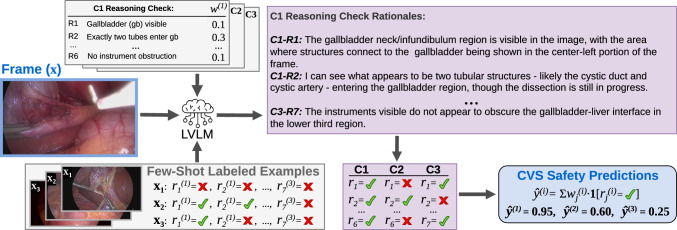
Pipeline for structured surgical reasoning with Sum-of-Checks. For each CVS criterion, we define a set of expert-aligned reasoning checks, each assigned a fixed weight reflecting its relative importance. Given a laparoscopic frame *x*, we prompt an LVLM to evaluate each check, producing a short justification and a binary judgment. Few-shot examples with labeled reasoning checks are also provided in the prompt context. Criterion-level scores $\hat{y}^{(i)}$ are then computed by aggregating the binarized check outcomes using a weighted sum

Machine learning models are increasingly deployed in high-stakes medical domains, where incorrect predictions can lead to irreversible harm [1, 2]. However, existing specialized systems and language models typically require substantial task-specific labeled data [3–7], limiting their ability to adapt to new clinical questions or changing safety requirements. Large vision-language foundation models (LVLMs) offer a more flexible alternative, leveraging large-scale pretraining to acquire medical knowledge and generalize across tasks [8, 9]. By jointly reasoning over both visual and textual information, they can support complex clinical prompts beyond single-label prediction.

However, large foundation models still struggle with certain clinically important tasks, particularly those requiring reasoning over diverse and interdependent visual evidence rather than simple recognition of isolated cues [8, 9]. One such task is assessing the Critical View of Safety (CVS) during laparoscopic cholecystectomy [10], which demands jointly evaluating anatomical visibility, spatial configuration, and the explicit exclusion of ambiguity [3, 4, 10–12]. Even when augmented with prompting strategies such as chain-of-thought, the resulting reasoning processes are difficult to systematically audit or constrain [13, 14], limiting clinicians’ ability to verify whether safety-critical evidence has been explicitly considered.

To address this gap, we propose **Sum-of-Checks**, a structured framework for surgical safety assessment that decomposes each safety criterion into expert-defined verification checks, shown in Fig. 1. We collaborate with surgeons to define clinically meaningful checks that reflect the evidence they routinely seek (e.g., anatomy visibility, spatial relationships, and observational reliability), and aggregate these check-level judgments into criterion-level scores using a fixed, interpretable weighting scheme. By explicitly separating evidence elicitation from final scoring, Sum-of-Checks leverages the medical knowledge encoded in LVLMs to predict structured clinical evidence, yielding more accurate safety assessments while enabling fine-grained inspection and targeted intervention when individual checks fail.

**Table 1:** Frame-level CVS assessment performance (mAP)

Model	Method	C1	C2	C3	Avg
GPT-4.1 mini	Direct	22.9$_{\pm 0.5}$	23.9$_{\pm 1.4}$	42.5$_{\pm 1.2}$	29.7$_{\pm 0.9}$
	Direct+FS	26.9$_{\pm 0.7}$	26.8$_{\pm 0.4}$	42.9$_{\pm 0.8}$	32.2$_{\pm 0.4}$
	CoT	26.2$_{\pm 0.6}$	27.7$_{\pm 1.7}$	43.9$_{\pm 0.8}$	32.6$_{\pm 0.6}$
	CoT+FS	26.9$_{\pm 1.0}$	28.2$_{\pm 0.4}$	44.3$_{\pm 0.9}$	33.1$_{\pm 0.4}$
	SubQ	22.5$_{\pm 1.2}$	25.9$_{\pm 0.8}$	43.4$_{\pm 1.2}$	30.6$_{\pm 0.8}$
	SubQ+FS	22.1$_{\pm 1.2}$	21.5$_{\pm 0.2}$	43.8$_{\pm 0.3}$	29.1$_{\pm 0.5}$
	**Sum-of-Checks** (ours)	**33**.**6**$_{\pm 0.8}$	**29**.**1**$_{\pm 1.0}$	**47**.**9**$_{\pm 1.1}$	**36**.**9**$_{\pm 0.5}$
Claude Haiku 4.5	Direct	20.7$_{\pm 0.3}$	20.8$_{\pm 0.2}$	42.2$_{\pm 0.2}$	27.9$_{\pm 0.1}$
	Direct+FS	21.1$_{\pm 0.2}$	21.9$_{\pm 0.7}$	46.5$_{\pm 0.8}$	29.8$_{\pm 0.4}$
	CoT	19.7$_{\pm 0.7}$	21.7$_{\pm 0.8}$	43.8$_{\pm 1.3}$	28.4$_{\pm 0.4}$
	CoT+FS	22.5$_{\pm 0.5}$	23.7$_{\pm 0.2}$	43.1$_{\pm 0.6}$	29.7$_{\pm 0.4}$
	SubQ	23.4$_{\pm 0.5}$	22.5$_{\pm 0.7}$	43.3$_{\pm 0.3}$	29.8$_{\pm 0.2}$
	SubQ+FS	22.4$_{\pm 0.3}$	23.8$_{\pm 0.7}$	42.5$_{\pm 0.3}$	29.6$_{\pm 0.4}$
	**Sum-of-Checks** (ours)	**29**.**2**$_{\pm 0.3}$	**24**.**2**$_{\pm 0.5}$	**48**.**5**$_{\pm 0.1}$	**34**.**0**$_{\pm 0.2}$
Claude Opus 4.5	Direct	24.3$_{\pm 0.4}$	25.4$_{\pm 0.1}$	42.9$_{\pm 0.5}$	30.9$_{\pm 0.3}$
	Direct+FS	28.0$_{\pm 0.2}$	26.7$_{\pm 0.2}$	43.9$_{\pm 0.7}$	32.9$_{\pm 0.3}$
	CoT	26.4$_{\pm 0.4}$	26.6$_{\pm 0.8}$	42.5$_{\pm 1.0}$	31.9$_{\pm 0.2}$
	CoT+FS	27.6$_{\pm 0.2}$	26.0$_{\pm 0.4}$	43.4$_{\pm 0.1}$	32.3$_{\pm 0.2}$
	SubQ	24.1$_{\pm 1.9}$	24.5$_{\pm 0.1}$	43.0$_{\pm 0.6}$	30.5$_{\pm 0.8}$
	SubQ+FS	19.2$_{\pm 1.1}$	19.7$_{\pm 1.6}$	43.9$_{\pm 0.5}$	27.6$_{\pm 0.9}$
	**Sum-of-Checks** (ours)	**31**.**1**$_{\pm 0.3}$	**32**.**9**$_{\pm 0.4}$	**46**.**9**$_{\pm 0.1}$	**37**.**0**$_{\pm 0.1}$

Bold indicates the best result for each criterion (column), showing that our final method achieves higher mAP than ablations where different components of the framework are replaced

Sum-of-Checks outperforms all baseline methods across three models in predicting all three Critical View of Safety (CVS) criteria on Endoscapes. Cells report mean average precision (mAP, %) as mean ± standard deviation over 3 independent runs with stochastic LLM decoding (temperature =0.1). Best results are bolded

Our contributions are threefold: (1) We develop **Sum-of-Checks**, a framework for structured surgical safety prediction that operationalizes expert-defined reasoning checks as explicit intermediate decision units. (2) We show that **Sum-of-Checks** consistently outperforms free-form reasoning, demonstrating that, even when supplemented by few-shot examples, explicitly structured decision processes are critical for reliable surgical reasoning. (3) We show that while LVLMs reliably predict observational reliability checks (i.e., camera exposure, instrument obstruction), their performance varies substantially on anatomical evidence checks, highlighting a key limitation in current model reasoning for surgical assessment.^1^

## Sum-of-Checks: structured surgical reasoning

The Critical View of Safety (CVS) is a surgical standard for preventing bile duct injury during laparoscopic cholecystectomy [10]. It requires satisfying three criteria: **Criterion 1 (C1)**: Two and only two tubular structures are visible entering the gallbladder. **Criterion 2 (C2)**: The hepatocystic triangle is cleared of fat and fibrous tissue. **Criterion 3 (C3)**: The lower third of the gallbladder is detached from the liver bed [3, 11]. Each criterion is assessed using visual evidence directly observable in individual laparoscopic frames.

### Problem setup

Let *x* denote a laparoscopic frame, and let $y^{(i)} \in \{0,1\}$ indicate whether criterion $i \in \{1,2,3\}$ is satisfied in that frame. Our goal is to predict $\hat{y}^{(i)} \in [0,1]$ for each criterion.

### Expert-defined reasoning checks

**Table 2:** Sum-of-Checks Ablation (GPT-4.1-mini)

Method	C1	C2	C3	Avg
Sum-of-Checks (no FS)	23.4$_{\pm 0.3}$	23.9$_{\pm 1.4}$	44.1$_{\pm 0.5}$	30.5$_{\pm 0.4}$
Sum-of-Checks (LLM agg.)	29.3$_{\pm 2.1}$	27.2$_{\pm 0.8}$	46.5$_{\pm 0.6}$	34.3$_{\pm 0.8}$
Sum-of-Checks (Weighted) (ours)	**33**.**6**$_{\pm 0.8}$	**29**.**1**$_{\pm 1.0}$	**47**.**9**$_{\pm 1.1}$	**36**.**9**$_{\pm 0.5}$

Bold indicates the best result for each criterion (column), showing that our final method achieves higher mAP than ablations where different components of the framework are replaced

**Fig. 2:**
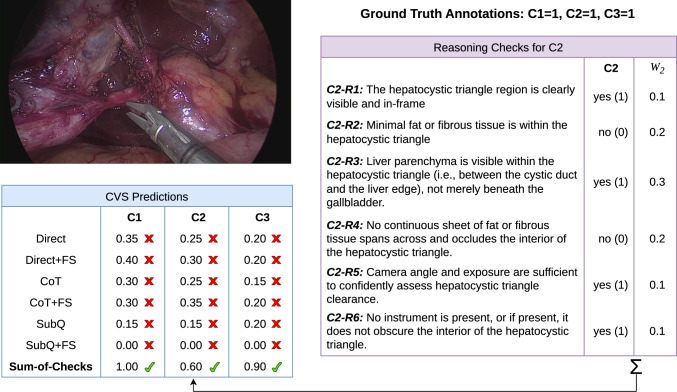
Example output of Sum-of-Checks (Claude Opus 4.5). All three CVS criteria are satisfied (ground truth = (1,1,1)). Standard prompting methods fail on all criteria, whereas Sum-of-Checks correctly predicts all three. For C2 (hepatocystic triangle clearance), the model identifies satisfied sub-checks (e.g., visibility of liver parenchyma, sufficient exposure), yielding a weighted score of 0.6>0.5 and a correct prediction

Rather than predicting each criterion in a single model call, Sum-of-Checks introduces an explicit intermediate representation that separates evidence elicitation from final decision-making. For each criterion *i*, we specify a set of expert-defined reasoning checks $\mathcal {R}^{(i)} = \{ R^{(i)}_j \}_{j=1}^{m^{(i)}}$, where $m^{(i)}$ denotes the number of checks associated with criterion *i*, and each $R^{(i)}_j$ is a natural-language verification condition routinely used by surgeons when assessing CVS. These checks capture key aspects of expert reasoning, including anatomical visibility, spatial configuration, exclusion of ambiguity, and control of occlusions.

Given a frame *x* and the full set of reasoning checks for criterion *i*, the LVLM produces binary scores

1 $$\begin{aligned} r^{(i)}_j = f_j(x, \mathcal {R}^{(i)}) \in \{0,1\}, \quad j = 1, \ldots , m^{(i)}, \end{aligned}$$

where $f_j$ denotes the composition of the LVLM and a fixed post-processing function that maps the model’s textual judgment for check *j* (e.g., *yes*, *no*, or *uncertain*) to a binary value, with affirmative responses assigned 1 and all other responses assigned 0, treating ambiguity as insufficient evidence for safety-critical decisions.

### Check aggregation

Criterion-level predictions are obtained by aggregating the scored reasoning checks. For each criterion *i*, we assign non-negative weights $\textbf{w}^{(i)} = (w^{(i)}_1, \dots , w^{(i)}_{m^{(i)}})$ such that $\sum _{j=1}^{m^{(i)}} w^{(i)}_j = 1$. The final prediction is computed as a weighted average:

2 $$\begin{aligned} \hat{y}^{(i)} = \sum _{j=1}^{m^{(i)}} w^{(i)}_j \, r^{(i)}_j. \end{aligned}$$

Both the reasoning checks and their weights are defined in collaboration with expert surgeons. This aggregation scheme enables transparent inspection of how individual checks contribute to the final judgment.

## Experiments

### Experimental setup

We evaluate Sum-of-Checks on Endoscapes2023 [3], a benchmark for frame-level CVS assessment in laparoscopic cholecystectomy, using a randomly selected half of the official test split (791 frames) with 4 few-shot examples. We test on three frontier models: GPT-4.1-mini [15], Claude Haiku 4.5 [16], and Claude Opus 4.5 [17].^2^ We compare against: *Direct* prompting, *CoT* [18] (free-form chain-of-thought reasoning), and *SubQ* (model-generated QA pairs used as intermediate reasoning before prediction), each with and without few-shot examples (+FS). Frame-level mean average precision (mAP) is reported in Table 1.

### Sum-of-Checks improves CVS assessment compared to unstructured prompting

As shown in Table 1, Sum-of-Checks consistently outperforms all baseline prompting techniques across all three criteria and all LVLMs. Standard CoT and SubQ provide only marginal or inconsistent improvements over Direct prompting, and adding few-shot examples (+FS) yields limited gains for these methods, suggesting that neither free-form reasoning nor exemplar guidance alone reliably improves CVS assessment. In contrast, Sum-of-Checks effectively leverages both structured checks and few-shot examples to achieve substantial improvements.

### Few-shot exemplars and weighted average are both essential for Sum-of-Checks

Table 2 shows that removing few-shot exemplars leads to a substantial performance drop, indicating that structured checks alone are insufficient—mirroring the behavior of SubQ, where the absence of strong exemplar guidance limits reliable prediction. Replacing weighted aggregation with LLM-based aggregation also degrades performance, suggesting that deterministic weighted aggregation provides more stable integration of check-level signals than an additional LLM reasoning step.

## Discussion and conclusion

Sum-of-Checks substantially improves frame-level CVS assessment by decomposing safety criteria into expert-defined verification checks that explicitly separate evidence elicitation from decision-making, improving both accuracy and interpretability. Figure 2 illustrates this on a representative frame where all baselines fail but Sum-of-Checks correctly predicts all three criteria.

### Reasoning check prediction reliabilities

LVLMs’ performance in completing reasoning checks varies substantially across check types, even within the same criterion. Observational checks—such as camera exposure, field of view, and instrument obstruction—are predicted consistently, while checks requiring anatomical evidence and spatial reasoning exhibit much greater variability. This indicates that current LVLMs remain less reliable on the decision-critical evidence required for complex CVS assessments.

### Few-shot exemplar selection

Randomly selected exemplars are insufficient for reliable predictions. We select 4 diverse examples with label combinations (000, 111, 110, 001) and find two properties of effective exemplars: (1) *typicality*—clear, canonical instances of each condition; and (2) *minimal variation*—paired exemplars differing in only one criterion should share the same visual context. Our results depend on in-distribution exemplars; we leave out-of-distribution cases to future work.

## Limitations

We evaluate on Endoscapes2023 [3], which provides expert-annotated key frames aligned with our frame-level, check-based evaluation protocol. Other datasets such as Cholec80-CVS [19] and the SAGES CVS Challenge [14] differ in annotation granularity or distribution; we leave these extensions, along with application to other safety-critical reasoning tasks, to future work.

## Footnotes

1 Code is available at https://github.com/BrachioLab/SumOfChecks

2 All models are accessed in February 2026.
